# The Recent Death in a Dentist's Chair

**Published:** 1874-04

**Authors:** J. F. Babcock


### ARTICLE V.

### *The Recent Death in a Dentist's Chair.*

J. F. Babcock, D. D. S., writes to the *Boston Medical and Surgical Journal*, as follows:

"It is evident to me that exceptions of the strongest nature may be taken to the wisdom and accuracy of the verdict recently returned by the jury, in the case of the late Mrs. Crie, who died while inhaling a mixture of ether and chloroform in the dental chair of Dr. Eastham, in Boston. The jury came to the conclusion that 'her death was caused by the inhalation of chloroform,' and took the opportunity to 'caution the public against the inhalation of so dangerous an agent as chloroform for the production of insensibility to pain.'

I have watched this case with exceeding care, and, if testimony of the witnesses may be relied upon, I fail to perceive the slightest foundation for such a verdict, or any reason, growing out of the investigation of this affair, which should warrant the sweeping condemnation of chloroform implied by the 'caution.' My exceptions to the verdict take form, when I assert my belief that there were three other distinct, and exceedingly probable, causes of death in this case, independent of *direct* death from the action of chloroform.

First, we have the testimony of Mrs. Sawyer, who was present during the operation, and who assisted in the endeavors to resuscitate the unfortuuate lady, that she 'never saw a woman so tightly laced in her life.' Please remember that a *woman* says this, and then imagine what must have been the effect upon Mrs. Crie's organs of respiration. She could not inhale the usual quantity of atmospheric air so very important to be freely mixed with the chloroform, nor could she exhale the normal quantity of carbonic acid gas so vitally requisite under such circumstances, while such *intense* compression would tend to crowd the lungs upon the heart, and impede, or perhaps, *wholly arrest its action*, especially were it a weak one. There are cases upon record where jurors have found, under exactly the same conditions, where chloroform, or any other anæsthetic, has been exhibited, and where death followed, that the result was due to the compression of the respiratory organs induced by tight lacing. I ask, is it not fair to suppose that Mrs. Crie's death *might* have been due to tight lacing and consequent undue compression of the lungs and heart?

Secondly, it is the testimony of several witnesses that Mrs. Crie was of what might be termed a hyper-nervous temperament. Her excessive fear that she might possibly feel pain from the operation, as she did upon a previous occasion, compelled her to refuse the administration of nitrous oxide, or laughing gas, and induced her to beg Dr. Eastham to be '*sure* and give her enough.' This request she repeated several times, while he assured her that he would do so. All this goes to demonstrate the excessive fear with which she regarded the operation, and which, in my opinion, should have contraindicated the exhibition of chloroform, unless such excessive fear could have been previously allayed. No person, save those who have witnessed it, can realize the effect of such extreme fear in women of a highly nervous and sensitive temperament. It is in the testimony that Dr. Eastham proceeded with the administration of the anæsthetic, and while she was evidently in a conscious condition, *said* to her, 'I am going to extract this tooth now,' to which she expressed decided disapproval, but he proceeded to carry his intention into execution, and did so, when she screamed with fear and pain, and immediately went into a spasm, from which she never recovered.

Up to the time of the extraction of the tooth, it is not mentioned that there were any contra-indications developed which forbade further administration of the anæsthetic; but instantly after the operation, she sank rapidly. As is well known, fear has a most depressing influence on the heart's action, and this should be carefully taken into consideration when administering chloroform. How many persons faint, even while preliminary arrangements, in anticipation of an operation, are going forward, and, indeed, how many are the cases upon record, of death from such fright. It would seem, then, but a reasonable supposition that Mrs. Crie's death might have been due to the depression of the heart's action, induced by excessive fright, combined with its inability to recover from such depression in consequence of the pressure of the lungs upon it, induced by the tight lacing.

Thirdly, it is in the evidence that the post mortem revealed the fact that the muscular walls of the heart were abnormal in their lack of tone, that they were weak, unusually so. Now, while the condition of this heart alone was sufficient, were it known beforehand, to constitute a contra-indication, yet, when you take into conjunction with it, the natural effect of fear, and the compression of the lungs upon it, is it at all strange that Mrs. Crie should have met with her death as she did? In fact, would it not have been most remarkable if she had lived? It seems to me that it would have been almost a miracle. In view of these facts, why make such a sweeping condemnation of chloroform? I would not be understood as being an advocate of its use, whenever it can be dispensed with, for it possesses a property, in that it is a local anæsthetic—which ether is not—which makes it more or less dangerous, especially so in abnormal conditions of the heart. Dip the finger in chloroform and it will become sufficiently insensible to pain to perform operations upon it. In all probability, fatal results arise from this very ability to produce local anæsthesia. The blood goes from the lungs directly to the left auricle of the heart, thence to the left ventricle to be propelled through the aorta. The cardiac arteries going to, and supplying, the heart's structure, charged with chloroform vapor, which may cause local anæsthesia of the heart, and hence cessation of its action, or syncope; the heart's muscular fibres are relaxed and deadened, and thus it follows the chloroform is, so far as the heart is concerned, a specific narcotic, and hence its danger. But in some operations, especially in the oral cavity, where the first step may be of a character starting, in some sustances, frightful hæmorrhage, which can be combatted only at the completion of operation; to have a patient pass from control at such a moment, and under such circumstances (as they are most assuredly liable to do with ether alone), is sometimes a matter of serious concern; and, under such conditions, I maintain that chloroform is indispensable, and those gentlemen who composed that jury—if it be that they were any of them practitioners of surgery—known it just as well as I do, and, therefore, I ask again why such an *unqualified* condemnation of chloroform? Had they restricted their disapproval to its inhalation for the performance of so simple an operation as the extraction of a tooth, I should have agreed with them heartily. But they did not; they caution the public "against the inhalation of so dangerous an agent as chloroform for the production of insensibility to pain," regardless of the character of the operation to be performed. Such a condemnation is unjust in every sense of the word, since it condemns in advance every surgeon who deems it his duty to administer it, and doubly so when its foundation suggested by the results in Mrs. Crie's case (where the only reason they had, as it would seem, was the fact that she inhaled it), as it has been the aim of this article to demonstrate.

[We readily publish this letter as a proof of our willingness to hear both sides of the question ; but we cannot attach any importance to the objections of our correspondent, with the exception, perhaps, of the first. We do not know on what authority he states that Mrs. Sawyer testified that she "never saw a woman so tightly laced in her life ;" according to our special reporter, she said only that the deceased was laced very tightly. With regard to the intense fear, it is entirely an assumption : there is no evidence to show it. The fact that the patient asked thrice to have enough of the anæsthetic shows confidence rather than anxiety. The statement that chloroform is indispensable in severe operations in the mouth, and we may add, under any circumstances in cival practice, is contrary to all experience in cities where ether is understood and come only from a want of familiarity with the latter anæsthetic.—Eds.]

---

